# Wood Colorization through Pressure Treating: The Potential of Extracted Colorants from Spalting Fungi as a Replacement for Woodworkers’ Aniline Dyes

**DOI:** 10.3390/ma7085427

**Published:** 2014-07-24

**Authors:** Sara C. Robinson, Eric Hinsch, Genevieve Weber, Kristina Leipus, Daniel Cerney

**Affiliations:** 1 Department of Wood Science & Engineering, 119 Richardson Hall, Oregon State University, Corvallis, OR 97331, USA; E-Mails: eric.hinsch@oregonstate.edu (E.H.); genevievelweber@gmail.com (G.W.); 2 School of Forest Resources and Environmental Science, Michigan Technological University, 1400 Townsend Dr., Houghton, MI 49931, USA; E-Mails: kmleipus@mtu.edu (K.L.); djcerney@mtu.edu (D.C.)

**Keywords:** aniline dye, *Chlorociboria* spp., *Scytalidium* spp., spalting

## Abstract

The extracellular colorants produced by *Chlorociboria aeruginosa*, *Scytalidium cuboideum*, and *Scytalidium ganodermophthorum*, three commonly utilized spalting fungi, were tested against a standard woodworker’s aniline dye to determine if the fungal colorants could be utilized in an effort to find a naturally occurring replacement for the synthetic dye. Fungal colorants were delivered in two methods within a pressure treater—the first through solubilization of extracted colorants in dichloromethane, and the second via liquid culture consisting of water, malt, and the actively growing fungus. Visual external evaluation of the wood test blocks showed complete surface coloration of all wood species with all colorants, with the exception of the green colorant (xylindein) from *C. aeruginosa* in liquid culture, which did not produce a visible surface color change. The highest changes in external color came from noble fir, lodgepole pine, port orford cedar and sugar maple with aniline dye, cottonwood with the yellow colorant in liquid culture, lodgepole pine with the red colorant in liquid culture, red alder and Oregon maple with the green colorant in dichloromethane, and sugar maple and port orford cedar with the yellow colorant in dichloromethane. The aniline dye was superior to the fungal colorants in terms of internal coloration, although none of the tested compounds were able to completely visually color the inside of the test blocks.

## 1. Introduction

There is a long history of use of natural and synthetic colorants on wood products to enhance visual appeal, with the documentation of Pliny the Elder (23-79AD) as one of the earliest accounts of such wood modification [1]. The preferences of artists and consumers for various types of synthetic and natural colorants have changed substantially over time, with very few ancient methods still utilized today.

Two primary forms of colorants are utilized by modern woodworkers—modern “ease of use” aniline dyes, referring to powdered synthetic dyes that can be solubilized in alcohol or hydrated in water, and spalted wood, referring to wood that has been given color(s) through colonization of a very select group of fungi. Although modern aniline dyes bear little similarity to their historical 19th Century counterparts such as triphenylmethane dyes, the use of spalting for woodcraft dates back to at least the 15th Century [2] and there has been little change in usage techniques over time.

However, the past two decades have seen a growing interest in switching from modern aniline dye methods for coloring wood to the use of entirely spalted wood [3]. This shift has presented numerous problems for the woodworking community, as aniline dyes can be cheaply purchased and readily applied to finished or unfinished wood products with little effort. Spalted wood, on the other hand, must either be found in nature before the decay effects of the fungi have been fully realized, thus rendering the wood unusable, or the spalting must be induced in clear wood—a process that can take months to several years with unreliable results.

Laboratory research has managed to decrease the maximum incubation time necessary for inducing spalting to around twelve weeks [3]. However, this amount of time is still too long to appeal to many woodworkers used to the instant effects produced by aniline dyes. A new method has recently been developed to extract the colorants produced by some of the popular spalting fungi to produce a solution similar to that of an alcohol-solubilized aniline dye [4]. However, how this colorant solution moves through wood, the concentrations necessary to develop the same intensity of color as given by aniline dye, and the best method to achieve internal coloration have yet to be explored.

The aim for this research was to test a pressure treatment method to color wood with the solubilized colorant obtained from fungi used for spalting and compare the results to those gained with aniline dyes. If similar results can be achieved, woodworkers will have a comparable method for coloring wood that does not utilize synthetic colorants, and that maintains the use of an historic art form.

## 2. Experimental Section

### 2.1. Wood Selection

Darker woods were excluded due to the potential masking of color effects. Samples were cut into 14 mm cubes and kiln dried to 12% moisture content (MC). Samples were then allowed to equilibrate to ambient relative humidity for four weeks before testing (resulting MC of roughly 10%). Ten wood species were selected for testing based on their potential to show color change. Wood species utilized included: ash (*Fraxinus latifolia* Benth.), chinkapin (*Chrysolepis chrysophylla* Douglas ex Hook.), cottonwood (*Populus trichocarpa* Torr. & A.Gray), lodgepole pine (*Pinus contorta* Dougl. ex. Loud), noble fir (*Abies procera* Rehd.), Oregon maple (*Acer macrophyllum* Pursh), port orford cedar (*Chamaecyparis lawsoniana* (A. Murray)), red alder (*Alnus rubra* Bong.), sweet cherry (*Prunus avium* (L.)), and sugar maple (*Acer saccharum* Marsh.).

### 2.2. Fungal Selection

Three species of fungi were chosen due to their prolific extracellular colorant production and their frequent use in spalting: *Scytalidium cuboideum* (Sacc. & Ellis) Sigler & Kang (UAMH 4802, isolated from oak, location unknown, produces red/pink stain), *Chlorociboria aeruginosa* (Nyl.) Kanouse (UAMH 11657, isolated from a hardwood log in Ontario, ON, Canada, produces a blue-green colorant), and *Scytalidium ganodermophthorum* Kang, Sigler, (UAMH 10320, isolated from an oak log in Gyeonggi Province, Korea, produces yellow stain).

### 2.3. Colorants

#### 2.3.1. Isolation

Fungal colorants were gathered by two methods. The first was through growth of fungi in wood-agar plates as described in Robinson *et al.* [5], with extraction and resolubilization of colorants in dichloromethane (DCM) following the procedure outlined in Robinson *et al.* [4]. The second was through liquid culture growth, with the fungi grown on malt media prepared in half-pint Mason jars with plastic screw lids. Jars and lids were sterilized via autoclaving before use. After sterilization, jars were filled with 50 mL of autoclaved liquid media (2% malt in deionized water). Each jar was inoculated with five 6 mm plugs from an agar plate of an actively growing fungal culture species. Liquid cultures were stored on an open shelf at room temperature for six weeks. The media were then filtered using a Whatman Cat. No. 1002150 filter sheet and combined in a 1 L flask for each species. Color measurements were recorded on a Konica Minolta CR-5 chroma meter for each flask of media before use, with output in the CIE L*a*b* color space (S. cuboideum L* = 90.77, a* = 9.92, b* = 12.82; S. ganodermophthorum L* = 87.04, a* = −0.54, b* = 22.21; C. aeruginosa L* = 96.54, a* = −3.03, b* = 2.88).

#### 2.3.2. Concentration

Extraction solutions taken from the wood-agar plates were rehydrated at approximately 0.5 μg/mL (*C. aeruginosa*), 1 μg/mL (*S. cuboideum*) or 20 μg/mL (*S. ganodermophthorum*) by adding DCM to the dry state and allowing the colorant to resolubilize overnight. Resulting L*a*b* values were recorded on the chroma meter. All additional solutions, including the water solution, were matched to ±2.0 from these associated L*a*b* values (C. aeruginosa L* = 82.28, a* = −11.06, b* = −5.40; S. cuboideum L* = 82.32, a* = 26.84, b* = 13.19; S. ganodermophthorum L* = 95.46, a* = −3.00, b* = 8.15).

A blue-green colored aniline dye (brand: J.E. Moser’s) was dissolved in ethanol and diluted until the L* value matched (±2) the L* value of the wood-agar plate *C. aeruginosa* extract solution (L* = 84.11, a* = 18.62, b* = −21.90).

#### 2.3.3. Application onto Wood

Sixteen 250 mL flasks were prepared with solutions: four with the aniline dye, four each with the three fungal colorants in DCM solution extracted from wood plates, and four each with the fungal colorants in liquid culture as described above. Three replicates of each of the 10 wood species (30 blocks total) were distributed between the four flasks for each colorant solution and the blocks were weighted so they would not float. Colorant solution (75 mL) was added to each flask as appropriate. The total volume in each flask was 125 mL including wood blocks. The flasks were stoppered with foam rubber stoppers then placed in a pressure treating chamber. A vacuum of −27 HG was pulled for 30 min. Pressure was then applied at 100 psi for 30 min. After the pressure was released, the flasks were removed from the chamber. Blocks treated with aniline dye or fungal colorant in DCM were removed from the flasks and allowed to dry for 48 h, in ambient air under a fume hood. Liquid culture treated blocks were dried overnight at 65 °C.

#### 2.3.4. Assessment

After 48 h the blocks were assessed for color change on the chroma meter, using the readings taken before pressure treating as the baseline color. Control readings were taken on blocks treated with just DCM, just water, and just the liquid culture solution (*N* = 3). Blocks were then cut in half to expose an internal radial face and evaluated again using both the chroma meter for color change and ImageJ to determine the percent of the internal surface that showed color, using the protocol outlined in Robinson *et al.* [6].

Data were analyzed using SAS 9.3. External and internal color change was analyzed using a 3-way ANOVA with Tukey’s HSD, with wood species, test type, and color (red, green, yellow, aniline blue-green) as the dependent variables, and delta E* as the dependent variable. A two-way ANOVA was also run for both external and internal color change to differentiate results by wood species so that specific recommendations could be made for coloration method for each species.

To contrast the difference between what the human eye can discern as color change on various wood species *versus* what CIE Lab detects, a 2-way ANOVA was also run using internal percent surface coverage as determined by scanning as the dependent variable, by wood species. This was followed with Tukey’s HSD.

## 3. Results and Discussion

Control blocks treated with only DCM, only water, or only the liquid culture media showed an average of less than 1% change in their L*a*b* values between untreated and treated states on both external and internal faces.

### 3.1. External

**Figure 1:**
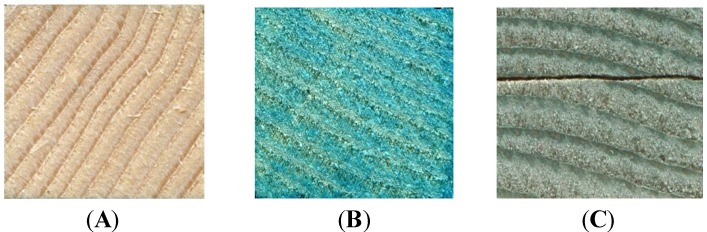
Coloring of external surface of test blocks. (**A**) Noble fir with no treatment; (**B**) aniline dye carried in ethanol on noble fir; (**C**) wood-agar colorant carried in dichloromethane (DCM) from *C. aeruginosa* on noble fir.

**Figure 2:**
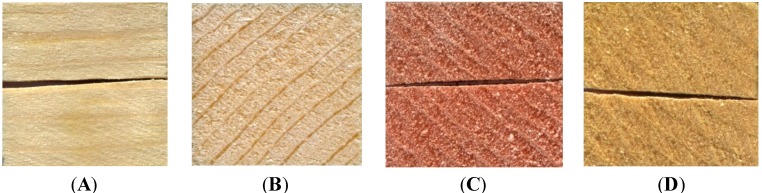
Coloring of external surface of test blocks from liquid cultures. (**A**) lodgepole pine with no treatment; (**B**) lodgepole pine with *C. aeruginosa*; (**C**) lodgepole pine with *S. cuboideum*; (**D**) lodgepole pine with *S. ganodermophthorum.*

In terms of visual color change, all treatments covered all surfaces of the test blocks, with the green colorant from *Chlorociboria aeruginosa* (Figure 1) being particularly striking. However, color change was much more apparent on the aniline dye blocks. The liquid cultures did not perform as well, with only the pink colorant of *S. cuboideum* and the yellow colorant of *S. ganodermophthorum* coloring all blocks, with lodgepole pine showing a strong color contrast (Figure 2). No visual external coloration occurred from liquid cultures of *C. aeruginosa*, although significant delta E* values did occur on red alder and Oregon maple, indicating that color change took place, but it was not sufficient to effect a visual color change.

The three-way ANOVA comparing the external color change for all four colorants (red, green, yellow, aniline dye) using L*a*b* data found all the independent variables significant at *p* < 0.0001 and all interactions significant at *p* < 0.0001 with the exception of the interaction of wood and color, which was significant at *p* = 0.0009. Despite the high significance, the Tukey HSD identified few significant differences. The noble fir blocks treated with aniline dye showed the greatest increase in delta E* values (558.15), but the value was not significantly different from sugar maple with aniline dye (525.19), sugar maple with yellow colorant in DCM (475.68), lodgepole pine with aniline dye (474.63), port orford cedar with aniline dye (415.07), lodgepole pine with red colorant in liquid culture (378.21), cottonwood with yellow colorant in DCM (368.13), red alder with green colorant in DCM, port orford cedar with yellow colorant in DCM (306.11) and Oregon maple with green colorant in DCM (302.87).

Although the aniline dye did produce significantly more external color change on the wood in most instances, it is important to note that all of the colorants were capable of changing the color of the test block surfaces, with the red and yellow colorants being the most versatile. The way the colorant was carried made a substantial difference, as no visual color change appeared on the surface of blocks pressure treated with the liquid culture of *C. aeruginosa*. The visual failure of *C. aeruginosa* in liquid culture was most likely due to the aggregation effect common with the green colorant xylindein [4]. At lower concentrations xylindein can be carried by many solvents, including water. However, there is a threshold at which the xylindein preferentially binds to either itself or the container and can only be mobilized with certain organic solvents. It is likely, due to the age of the liquid cultures, that the xylindein had already moved past the point of aggregation. Experiments with younger cultures may produce different results; however, this would also reduce the concentration of xylindein in solution.

In terms of external dyeing potential, the colorants produced by the fungi *S. cuboideum* and *S. ganodermophthorum* are just as efficient at surface coverage as a standard aniline dye of similar L* values (Table 1). Woodworkers and other craftspeople wishing to utilize natural, permanent dyes for their works should encounter few issues with using the extracted fungal colorants for surface dyeing of wood, especially if they pair the most effective coloration method with wood species. For example, very high delta E* values occurred in sugar maple and port orford cedar with yellow colorant in DCM, lodgepole pine with red colorant in liquid culture, cottonwood with yellow colorant in liquid culture, and red alder and Oregon maple with green colorant in DCM.

**Table 1:** ANOVA results for L*a*b* and percent coverage data by wood species. Different letters indicate statistically significant differences at alpha = 0.05 within each wood species and test column.

Wood	Method	Color	External Delta E*	Internal Delta E*	Internal % Coverage
Ash	aniline dye	blue-green	144.84 (A)	53.17 (A)	16.00 (A)
	liquid	red	168.89 (A)	25.59 (B)	0 (B)
		blue-green	22.68 (A)	18.16 (B)	0 (B)
		yellow	16.63 (A)	18.89 (B)	0 (B)
	wood-agar	red	100.40 (A)	1.57 (B)	0 (B)
		blue-green	100.40 (A)	11.22 (B)	0 (B)
		yellow	23.52 (A)	18.89 (B)	0 (B)
Chinkapin	aniline dye	blue-green	178.24 (A)	35.68 (A)	58.67 (A)
	liquid	red	128.94 (ABC)	10.18 (CD)	0 (B)
		blue-green	38.95 (E)	11.84 (CD)	0 (B)
		yellow	64.09 (CDE)	8.81 (D)	0 (B)
	wood-agar	red	134.84 (AB)	27.06 (BC)	0 (B)
		blue-green	110.52 (BCD)	27.06 (AB)	0 (B)
		yellow	53.38 (DE)	5.38 (D)	0 (B)
Cottonwood	aniline dye	blue-green	282.86 (A)	57.47 (B)	58.67 (A)
	liquid	red	264.71 (A)	0.76 (D)	0 (B)
		blue-green	64.71 (A)	2.39 (D)	0 (B)
		yellow	368.13 (A)	97.24 (A)	0 (B)
	wood-agar	red	131.78 (A)	29.62 (C)	2.9 (B)
		blue-green	99.47 (A)	36.61 (BC)	0 (B)
		yellow	45.49 (A)	1.43 (D)	0 (B)
Lodgepole pine	aniline dye	blue-green	474.46 (A)	65.26 (A)	55.33 (A)
	liquid	red	378.21 (B)	4.69 (C)	8.67 (B)
		blue-green	87.95 (D)	2.39 (C)	0 (B)
		yellow	185.69 (C)	3.79 (C)	0 (B)
	wood-agar	red	142.78 (CD)	16.94 (B)	0 (B)
		blue-green	113.85 (D)	24.94 (B)	0 (B)
		yellow	182.58 (C)	2.44 (C)	0 (B)
Noble fir	aniline dye	blue-green	558.15 (A)	46.96 (A)	52.33 (A)
	liquid	red	182.16 (B)	4.28 (B)	6.67 (B)
		blue-green	61.95 (B)	18.02 (B)	0 (B)
		yellow	110.23 (B)	5.84 (B)	0 (B)
	wood-agar	red	202.50 (B)	5.22 (B)	0.83 (B)
		blue-green	227.11 (B)	6.34 (B)	0 (B)
		yellow	160.88 (B)	2.79 (B)	8.57 (B)
Oregon maple	aniline dye	blue-green	162.74 (BC)	39.91 (A)	39.33 (A)
	liquid	red	136.04 (BC)	6.70 (B)	0.67 (B)
		blue-green	58.97 (C)	6.44 (B)	0 (B)
		yellow	70.05 (C)	5.61 (B)	0 (B)
	wood-agar	red	205.97 (AB)	31.85 (A)	0.57 (B)
		blue-green	302.87 (A)	30.76 (A)	0 (B)
		yellow	57.28 (C)	5.89 (B)	3.67 (B)
Port orford cedar	aniline dye	blue-green	415.10 (A)	61.75 (A)	60.00 (A)
	liquid	red	246.50 (A)	0.95 (D)	12.33 (B)
		blue-green	55.0 (A)	1.24 (D)	0 (B)
		yellow	112.10 (A)	1.73 (D)	0 (B)
	wood-agar	red	187.10 (A)	10.95 (C)	4.3 (B)
		blue-green	165.50 (A)	21.62 (B)	4.03 (B)
		yellow	306.10 (A)	3.50 (D)	9.33 (B)
Red alder	aniline dye	blue-green	142.76 (BC)	40.38 (B)	0 (A)
	liquid	red	78.27 (C)	8.59 (C)	0 (A)
		blue-green	78.27 (C)	8.37 (C)	0 (A)
		yellow	43.49 (C)	11.92 (C)	0 (A)
	wood-agar	red	259.61 (AB)	57.68 (A)	0 (A)
		blue-green	318.11 (A)	41.27 (B)	0 (A)
		yellow	150.56 (ABC)	5.61 (C)	1.33 (A)
Sweet cherry	aniline dye	blue-green	153.23 (B)	90.35 (A)	8.33 (A)
	liquid	red	63.87 (C)	11.36 (BC)	0 (B)
		blue-green	45.34 (C)	10.73 (BC)	0 (B)
		yellow	32.11 (C)	9.30 (C)	0 (B)
	wood-agar	red	241.47 (A)	94.77 (A)	0 (B)
		blue-green	193.19 (AB)	53.88 (AB)	0 (B)
		yellow	50.48 (C)	20.73 (BC)	0 (B)
Sugar maple	aniline dye	blue-green	525.19 (A)	52.94 (A)	12.00 (B)
	liquid	red	158.52 (B)	17.61 (BC)	10.67 (B)
		blue-green	49.53 (B)	12.60 (CD)	0 (C)
		yellow	29.53 (B)	15.42 (CD)	0 (C)
	wood-agar	red	218.02 (B)	9.94 (D)	0 (C)
		blue-green	201.77 (B)	12.43 (CD)	0 (C)
		yellow	475.68 (A)	23.10 (B)	18.33 (A)

Method = the process by which the colorant was applied to the wood; Color = the color of the colorant applied to the wood; Values that share a common letter are not statistically different from each other.

### 3.2. Internal

Internal results were much more variable than external results. Pressure treatment of any chemical into wood has challenges that can increase depending upon the unique anatomy of each wood species. Generally, species with low heartwood, few extractives, and large earlywood vessels can be pressure treated much more easily than those with more cellular blockage. Additionally, compounds that aggregate are far less likely to be pressure treated into wood, as more aggregation leads to larger compounds that cannot move as readily through wood. Fungi selected for this experiment produce colorants specific to their role in nature—to thrive inside wood through the accumulation of their anti-fungal colorants within the wood structure [7,8,9,10]. It was assumed that the aniline dyes would show much greater penetration, as the fungal colorants are meant to be delivered by the fungal hyphae or in very small concentrations through the wood via water conduction. In the case of visual internal color change this assumption was confirmed. All wood species showing more internal visual color from the aniline dye blocks than with the other treatments. The notable exception is sugar maple where there was significantly more visual internal color from the yellow colorant in DCM than from the aniline dye (Table 1). Why sugar maple was the only species to have significant visual internal color not from aniline dye is unknown. However, every laboratory spalting test to date has found sugar maple to be the superior wood species for spalting visual color changes [3]. When growing fungi directly on the wood this is attributed to the higher sucrose content of sugar maple [11]. However, as no live cultures were utilized in this experiment, it is unknown why the yellow colorant carried in DCM performed so well.

Interestingly, the results presented in Table 1 show that in some instances, the fungal colorants are able to create a significant color change; however, that color change may not be visible to the naked eye. The three-way ANOVA for internal color change was highly significant, with all independent variables and interactions significant at *p* < 0.0001 except color, which was significant at *p* = 0.0011. The highest delta E* values came from cottonwood treated with the liquid culture yellow (97.24), which had an internal visual surface yellow coverage of 0%, and sweet cherry pressure treated with red (94.77) that also showed no visual color change. The delta E* values of these two were not significantly higher than sweet cherry treated with aniline dye (90.35), which showed a visual percent coverage of 8.33%.

In the case of the sweet cherry, it is likely that the red color change was masked visually by the natural red color of the wood. The same may also be true of the cottonwood, which is naturally a pale yellow to white. Enlarged scanned images of the blocks with high delta E* readings but no visual color change (increased from 14 mm to 15 cm) revealed small flecks of the target color within the wood, all sufficiently separated so that a gross color change could not be visualized on the small sample. While this confirms the presence of the colorant and validates the larger delta E* readings it does not address the needed results—that a color change take place on the wood that is visual even on small areas. Hence, there is a need for a threshold of delta E* at which visual color changes can be discerned.

Different thresholds have been used to determine how much change in delta E* constitutes a visual color change, with companies responsible for matching paint colors to consumer samples using as low as delta E* = 1.0 as the maximum score before color change is visually detectible [12]. It is obvious from this research that a much higher threshold needs to be set for visual color changes to be seen in wood, taking into account the high color variability even within the same wood species, and the potential desire to increase the color saturation of a given species (turning a red wood “more red”, for example).

### 3.3. Comparing Percent Coverage to CIE Lab Values

To determine the delta E* threshold at which visual color change can be discerned, the internal block faces that received a “0” for percent internal coverage were also analyzed using the chroma meter. As the delta E* values and corresponding visual color change varied so widely with species, thresholds were set for each species individually. For each wood species, the lowest internal visual color coverage numbers was noted, as was its associated delta E* value. If there were no higher delta E* values within that wood species showed 0% internal color coverage, the value was recorded as the threshold value in Table 2. If a higher delta E* value was found that did not have an associated internal color coverage, the next highest internal visual color coverage number was used until the point was found at which there were no higher delta E* values without corresponding internal color coverage numbers. Thresholds were identified as the lowest delta E* value corresponding to a >1% visual internal coverage for the given species. Hypothetically, delta E* values at or above these species-specific thresholds will be associated with a visual color change.

**Table 2:** Minimum delta E* values required for a visual color change, by wood species.

Wood	Threshold (Delta E*)
ash	54
chinkapin	36
cottonwood	58
lodgepole pine	66
noble fir	47
Oregon maple	40
port orford cedar	22
red alder	NA
sweet cherry	91
sugar maple	18

NA, no internal color: no internal coloration occurred on any of the test samples from this wood species.

Table 2 provides a useful baseline guide of the lowest delta E* value required for a visual color change by wood species, regardless of colorant utilized. However, due to the highly variable nature of wood, these values may not routinely represent minimum delta E* values. Additionally, given the potential for differences in anatomy for each wood block, delta E* may not be an ideal measurement of internal color change for pressure treated woods.

## 4. Conclusions

Extracellular colorants extracted from the fungi *C. aeruginosa*, *S. cuboideum* and *S. ganodermophthorum* can be utilized as natural alternatives to woodworkers’ aniline dyes for the purpose of surface coloration of wood. The better carrier for these colorants differs by colorant, with both methods suitable for *S. ganodermophthorum* (with the exception of sugar maple), DCM generally better for *C. aeruginosa* (with no difference between treatments for ash, cottonwood, lodgepole pine, noble fir, port orford cedar, and sugar maple), and the best carrier varying by wood species with *S. cuboideum* (DCM: red alder, sweet cherry; liquid culture: lodgepole pine; all other species: no difference). Internal coloration does not work as well with the fungal colorants as it does for the tested aniline dye, with most significant color changes as measured by delta E* not appearing as visual color changes. To effectively use these extracellular fungal colorants for internal wood coloration, alternative methods of delivery should be researched. It is possible that a drip application with the colorants solubilized in DCM might be more effective at delivering the colorants into the wood than by pressure treating.
